# *Tinospora cordifolia* (Willd.) Hook. f. and Thoms. *(Guduchi)* – validation of the Ayurvedic pharmacology through experimental and clinical studies

**DOI:** 10.4103/0974-7788.64405

**Published:** 2010

**Authors:** Avnish K. Upadhyay, Kaushal Kumar, Arvind Kumar, Hari S. Mishra

**Affiliations:** *Department of Ayurved Research and Development, Patanjali Yogpeeth, Haridwar, India*; 1 *Patanjali Herbal Garden and Agrotech Department, DYMT (SIROs), Patanjali Yogpeeth, Haridwar, India*; 2 *Patanjali Biotech, Patanjali Ayurved Limited, Haridwar, India*; 3 *Department of Dravya Guna, Govt. Ayurvedic College, Gurukul Kangri, Haridwar, India*

**Keywords:** Ayurveda, potential herb, reverse pharmacology, *Tinospora cordifolia*

## Abstract

*T. cordifolia (Guduchi)* is a large, glabrous, perennial, deciduous, climbing shrub of weak and fleshy stem found throughout India. It is a widely used plant in folk and Ayurvedic systems of medicine. The chemical constituents reported from this shrub belong to different classes, such as alkaloids, diterpenoid lactones, glycosides, steroids, sesquiterpenoid, phenolics, aliphatic compounds and polysaccharides. Various properties of *T. cordifolia*, described in ancient texts of Ayurveda, like *Rasayana, Sangrahi, Balya, Agnideepana, Tridoshshamaka, Dahnashaka, Mehnashaka, Kasa-swasahara, Pandunashaka, Kamla-Kushta-Vataraktanashaka, Jwarhara, Krimihara, Prameha, Arshnashaka, Kricch-Hridroganashak*, etc., are acquiring scientific validity through modern research adopting "reverse pharmacological" approach. Potential medicinal properties reported by scientific research include anti-diabetic, antipyretic, antispasmodic, anti-inflammatory, anti-arthritic, antioxidant, anti-allergic, anti-stress, anti-leprotic, antimalarial, hepato-protective, immuno-modulatory and anti-neoplastic activities. This review brings together various properties and medicinal uses of *T. cordifolia* described in Ayurveda, along with phytochemical and pharmacological reports.

## INTRODUCTION

**Figure 1:**
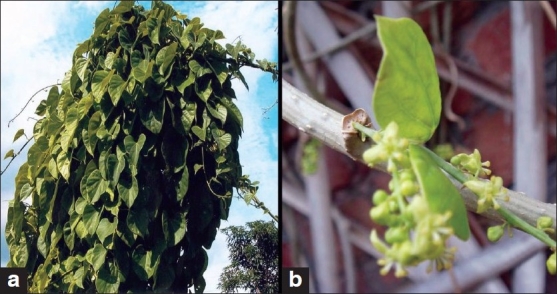
*Tinospora cordifolia* (a) Plant habit, (b) A view of stem with staminate and pistillate flowers

*Tinospora cordifolia* (Willd.) Hook. f. and Thoms. *(Guduchi)* is a large, glabrous, deciduous climbing shrub belonging to the family Menispermaceae.[1-3] It is distributed throughout the tropical Indian subcontinent and China, ascending to an altitude of 300 m. In Hindi, the plant is commonly known as *Giloe*,[4] which is a Hindu mythological term that refers to the heavenly elixir that has saved celestial beings from old age and kept them eternally young. Other common names and synonyms are *Guduchi, Amrita, Amritavalli, Madhuparni, Guduchika, Chinnobhava, Vatsadani, Tantrika, Kundalini, Chakralakshanika* (Sanskrit), *Gulancha* (Bengali), *Gurcha* (Hindi), *Garo, Galac* (Gujarati), *Thippateega* (Telugu), *Amrutavalli* (Kannada), *Amrita, Gilo* (Kashmiri), *Chittamrutu* (Malayalam), *Gulvel* (Marathi), *Guluchi* (Oriya), *Gilo* (Punjabi), *Seendal, Seendil Kodi* (Tamil), *Siddhilata, Amarlata* (Assamese) Heartleaf Moonseed, Tinospora (English).[5] *Guduchi*, the Sanskrit name, means one which protects the entire body. The term *amrita* is attributed to its ability to impart youthfulness, vitality and longevity. The stems of *T. cordifolia* are rather succulent with long filiform fleshy aerial roots from the branches. The bark is creamy white to gray, deeply left spiraily the space in between being spotted with large rosette-like lenticels. The leaves are membranous and cordate. The flowers are small and yellow or greenish yellow. In axillary and terminal racemes or racemose panicles, the male flowers are clustered and female flowers are usually solitary. The drupes are ovoid, glossy, succulent, red and pea sized. The seeds are curved. Fruits are fleshy and single seeded. Flowers grow during summer; and fruits, during winter.[6,7] Stem of the *T. cordifolia* appears in varying thicknesses, ranging from 0.6 to 5 cm in diameter; young stems are green with smooth surfaces and swelling at nodes, while the older ones show a light brown surface marked with warty protuberances due to circular lenticels; transversely smoothened surface shows a radial structure with conspicuous medullary rays traversing porous tissues; tastes bitter [Figure 1].[5]

## AYURVEDIC PHARMACOLOGY *(DRAVYA GUNA-KARMA*) OF *T. CORDIFOLIA (GUDUCHI)*

Ayurvedic pharmacology is based on biophysical, experiential, inferential and intuitional mechanisms. The action of a substance is based on five mechanisms of action or attributes of a substance, namely, *rasa* (taste appreciation of the substance by the chemical receptors on the tongue — Six tastes are described namely sweet (*madhura*), sour (*amla*), salty (*lavana*), bitter (*tikta*), pungent (*katu*)and astringent (*kasāya*), *guna* (10 pairs of opposite or mirror image attributes; attribute or property of any substance), *vipaka* (intestinal digestion and tissue metabolism; *madhura*- neutral, *amla*- acidic, *katu*- alkaline), virya (potency; *ushna*- hot, *sheeta*- cold) and *prabhav* (specific action through specialized receptors). All these mechanisms related to drug action are biophysical in nature. *Karma* is the action that involves the activity or performance. It is the final effectof the drug. The properties, action (pharmacodynamics) and uses (indication) of *T. cordifolia* are given in Table 1[5-8] and Table 2.[9-21] In the classical texts of Ayurveda, namely, *Charak, Sushruta* and *Ashtang Sangraha* and other treatises like *Bhava Prakash* and *Dhanvantari Nighantu*, etc., *T. cordifolia* is claimed to be useful in treating leprosy, fever, asthma, anorexia, jaundice, gout, skin infections, diabetes, chronic diarrhea, dysentery, etc.[9-21]

**Table 1:** Ayurvedic properties (*dravya-guna*) of *T. cordifolia* (*Guduchi*)[5-8]

*Rasa*	*Guna*	*Virya*	*Vipaka*	*Prabhava*
*Tikta, Kasaya*	*Laghu, Guru, Snigdha*	*Ushna*	*Madhura*	*Vishaghna*
Bitter, Astringent	Light, Heavy, Unctuous	Hot potency	Neutral	Anti-toxic

Rasa: Taste appreciation of the substances by chemical receptors on tongue; sweet, sour, salt, bitter, pungent and astringent, Guna: Ten pairs of opposite or mirror image attributes; attribute or property of any substance, Virya: potency; ushna- hot, sheeta- cold, Vipaka: Intestinal digestion and tissue metabolism; madhura- neutral, amlaacidic, katu- alkaline, Prabhava: Specific action through specialized receptors

**Table 2:** *Karma* (action - pharmacodynamics) and *prayoga* (uses) of *T. cordifolia*[9-21]

*Karma* (Action - Pharmacodynamics)	*Prayoga* (Uses- Indication)	Classical references
*Rasayana, Sangrahi, Balya, Agnidipana, Tridoshshamaka*	*Daha, Meha, Kasa, Pandu, Kamla, Kushta, Vatarakta, Jwara, Krimi, Prameha, Swas, Arsha, Kricch, Hridroga*	Bhav Prakash Nighantu,[9] Guduchyadi Varga; 8-10
*Vata-Pitta-Kaphanashak, Trishnanashaka, Agnideepaka*	*Jwara, Chardi, Daha*	Astang Sangrah[10] Sutrasthan 7-149, 16-10
*Sangrahi, Vatahara, Agnideepana, Shlesm-Shonit- Prashamana*	*Vivandha*	Charak samhita[11] Sutrastan 25-40
*Tridoshnashaka, Vishaghni, Jwara-bhootaghni*	*Jwara, Daha, Trishna, Vatarakta, Prameha, Pandu, Bhrama, Balipalita*	Raj Nighantu[12] Guduchyadi Varga 17,18
*Dipana, Grahi*	*Kasa, Pandu, Jwara*	Ark Prakash[13] Tritiya Shatak
*Balya, Tridoshnashaka*	*Laghujwara, Meha, Daha, Kasa, Pandu, Vitsarana (Atisara)*	Siddh Bhesajya Mani Mala[14] Dwitiya gucch 70
*Tridoshghni, Grahi, Rasayana, Dipana*	*Jwara, Daha, Kamla, Vatarakta*	Shodhal Nighantu[15] Guna Sangrah, Guduchyadi Varga-120
*Sangrahi, Balya, Agnideepana*	*Kamla, Kushta, Vatarakta, Jwara, Pitta, Vivandha, Krimi*	Madan Pal Nighantu[16] Harityakadi Varga-39,40,41
*Sangrahi, Vrishya, Balya, Rasayana, Dipana, Chakshusya, Vayah-Sthapana, Medhya, Tridoshanashaka*	*Kushta, Krimi, Chardi, Daha, Vatarakta, Pandu, Jwara, Kamla, Meha, Trishna, Kasa*	Kaidev Nighantu[17] Aushadhi Varga-9,10,11
*Tridoshanashaka, Aayushyaprada, Medhya, Sangrahi*	*Jantu, Raktarsha, Raktavata, Kandu, Visarpa, Kushta, Visha, Bhoota, Valipalita, Chardi, Meha, Jwara,*	Dhanvantari Nighantu[18] Guduchyadi- 5,6,7,8
*Grahi, Balya, Rasayana, Dipana, Hriddhya, Aayushyaprada, Chakshusya, Tridoshaghna*	*Jwara, Chardi, Kamla, Daha, Trisha, Bhrama, Pandu, Prameha, Kasa, Kushta, Krimi, Vatarakta, Kandu, Meda, Visarpa, Aruchi, Hikka, Arsha, Mutrakriccha, Pradara, Somroga*	Shaligram Nighantu[19] Guduchyadi Varga-251,252,253
*Pitta-Kaphapaha*	*Vataja Granthi, Vataja Galganda*	Sushrut Samhita, Sutra 46:270,[20] Chiki. 18: 5, 46[21]

## ETHNOBOTANICAL, FOLK AND TRIBAL USES OF *T. CORDIFOLIA (GUDUCHI)*

There are over 400 different tribal and other ethnic groups in India. Each tribal group has its own tradition, folk language, beliefs and knowledge about use of natural resources as medicines.[22] *T. cordifolia* finds a special mention for its use in tribal or folk medicine in different parts of the country. Some of the important uses reported in the literature[22] are listed in the Table 3. Almost all the parts of the plant are documented to be useful in ethnobotanical surveys conducted by ethnobotanists.[23-26]

**Table 3:** Uses of *T. cordifolia* (*Guduchi*) in folk and tribal medicine[22]

Tribals and areas	Diseases	Mode of application
Baiga, living in the interior areas of Naugarh and Chakia blocks of Varanasi district, Uttar Pradesh	Fever	The pills are prepared from the paste of stem of the *Guduchi (T. cordifolia)* and the roots of *Bhatkatiaya (Solanum surattense)*.
The tribals of Mumbai and its neighboring areas and the fi shermen along the sea cost	Fever, jaundice, chronic diarrhea, periodic fever	The whole plant is used.
The tribals of Khedbrahma region in north Gujarat	Cancer, dysentery, diarrhea and periodic fever	Powdered root and steam bark of *T. cordifolia* with milk for cancer.
		Decoction of root for dysentery and diarrhea. Decoction of old stems for periodic fever.
Tribals of Jammu ( J and K) and Bigwada (Rajasthan)	Fever	Decoction of stem is administered orally.
The inhabitants of Bhuvneshwar (Orissa)	Fever	The warm juice of root of *T. cordifolia* orally.
Inhabitants of Banka (Bihar)	*Balashosha* (Emaciation in children), *daha* (burning)	Dyed shirt soaked in juice of *Guduchi* worn by children for *Balashosha*.
		Paste or juice of *Amrita (T. cordifolia)* leaves and *Sarsapa beeja churna* (seed powder of *Brassica campestris)* are used for *daha*.
Local people of Patiala (Punjab)	Fever	Juice or decoction of leaves is administered orally with honey.
The Muslim tribals of Rajouri, Jammu (Tawi) comprising Gujjars and Backwals	Bone fracture	Whole part is used.
In Dhanu forest division of Maharashtra, tribal races, viz., Agaris, Bhils, Dhodias, Dublas, Khakaris, Rimoshis, Thakurs, Vandaries, Vagharis and Varlis	General debility	Decoction of stem with cold and hot water (about 3-4 g) in morning in an empty stomach, as a tonic.
People of Dhurala (Haryana)	*Kasa* (cough)	Powder of *Terminalia chebula (Haritiki), Tinospora cordifolia (Amrita)* and *Trachyspermum ammi (Ajwain)* in equal quantity is administered orally once daily early morning with salt.
Local people of Patiala (Punjab)	*Karna shula* (pain in ear)	Two drops of juice of leaves of allied species or *Guduchi (T. sinensis)* are dropped in the aff ected ear.
Local women of Arjunpura (Rajasthan)	*Raktapradara* (leukorrhea)	Paste of *Guduchi (T. cordifolia)* and 5 seeds of *Krishnamarich (Piper nigrum)* is administered orally daily in morning.
The inhabitants of Badala (UP)	*Swasa* (Asthma)	Juice of stem orally with honey.
People of Dehrabara Kolaras, Sivpuri District of M.P.	*Twak-roga* (Skin disease)	Decoction of stem is administered orally.
Mundas of Chhota Nagpur	Fracture	Paste of whole plant used as plaster.
In certain parts of India	Bites of poisonous insects and venomous snake, eye disorders.	The paste of *Guduchi* is applied to the part bitten and administered internally through mouth at intervals of half an hour.
		Juice or decoction of the root is poured into the eyes.

## PHYTOCHEMICAL CHARACTERIZATION

A large number of chemicals have been isolated from *T. cordifolia*, belonging to different classes such as alkaloids, diterpenoid lactones, glycosides, steroids, sesquiterpenoid, phenolics, aliphatic compounds and polysaccharides. Leaves of this plant are rich in protein (11.2%), calcium and phosphorus.[27] Four new clerodane furano diterpene glucosides (amritosides A, B, C and D) have been isolated as their acetates from stems. The structures of these compounds were established on the basis of spectroscopic studies.[28] The glycosyl component of a polysaccharide from *T. cordifolia* has been isolated, purified, methylated, hydrolyzed, reduced and acetylated. The partially methylated alditol acetate (PMAA) derivative thus obtained have been subjected to Gas Chromatography-Mass Spectrometry (GC-MS) studies. The following types of linkages were reported: terminal-glucose, 4-xylose, 4-glucose, 4, 6-glucose and 2, 3, 4, 6-glucose.[29,30] Callus and cell suspension cultures have been established from the stem explants of the plant. Accumulation of berberine and jatrorrhizine (protoberberine alkaloids) was observed in both callus and cell suspension cultures.[31] The signaling mechanism of the novel (1, 4)-alpha-D-glucan (RR1) isolated from *T. cordifolia* was investigated in macrophages to evaluate its immunostimulating properties.[32] An arabinogalactan has been isolated from the dried stems and examined by methylation analysis, partial hydrolysis and carboxyl reduction. Purified polysaccharide showed polyclonal mitogenic activity against B-cells; their proliferation did not require macrophages.[33] Detailed chemical constitution of *T. cordifolia* is given in Table 4.[34] Phytochemical characterization includes a test for one of the phytochemical components, namely, tinosporaside (limits, 0.03% to 0.04%).[35,36]

**Table 4:** Chemical constituents of *T. cordifolia* (*Guduchi*)[34]

Type of chemical	Active principles	Part in which present
Alkaloids (*Tikta*-Bitter Principle)	Berberine, Palmatine,	Stem
	Tembetarine, Magnofl orine,	Root
	Choline, Tinosporin, Isocolumbin, Palmatine, Tetrahydropalmatine, Magnofl orine	
Glycosides	18-norclerodane glucoside, Furanoid diterpene glucoside, Tinocordiside,	Stem
	Tinocordifolioside,	
	Cordioside, Cordifolioside A, Cordifolioside B,	
	Syringin, Syringin-apiosylglycoside,	
	Palmatosides C, Palmatosides F,	
	Cordifoliside A, Cordiofoliside B,	
	Cordifoliside C, Cordifoliside D, Cordifoliside E	
Diterpenoid lactones	Furanolactone,	Whole plant
	Clerodane derivatives and	
	[(5R,10R)-4R-8R-dihydroxy-2S-3R:15,16-	
	diepoxy-cleroda-13 (16), 14-dieno-17,12S:	
	18,1S-dilactone] and Tinosporon,	
	Tinosporides, and,	
	Jateorine, Columbin	
Steroids	β -sitosterol, δ-sitosterol, 20 β-	Aerial part
	Hydroxy ecdysone.	Stem
	Ecdysterone, Makisterone A,	
	Giloinsterol.	
Sesquiterpenoid	Tinocordifolin.	Stem
Aliphatic compound	Octacosanol, Heptacosanol,	Whole plant
Miscellaneous	Nonacosan-15-one	Whole plant
	3,(α,4-di hydroxy-3-methoxy-benzyl)-4-(4-	Root
	Compounds hydroxy-3-methoxy-benzyl)-tetrahydrofuran.	Whole plant
	Jatrorrhizine.	
	Tinosporidine, Cordifol, Cordifelone,	
	N-trans-feruloyl tyramine as diacetate,	
	Giloin, Giloinin, Tinosporic acid.	

## REVERSE PHARMACOLOGICAL AND CLINICAL CORRELATES

Indian contributions to the therapeutic revolution through reverse pharmacology will have to eventually integrate state-of-the-art high-throughput screening, combinatorial chemistry and effects of the old or novel compounds/ plants on human gene expression and proteomics.[37]

Much work has been done on *T. cordifolia* to validate its effects and this section describes some of these studies.

*T. cordifolia* has been used in Ayurvedic preparations for the treatment of various ailments throughout the centuries. It is used as a *rasayana* to improve the immune system and body resistance against infections. The whole plant is used medicinally; however, the stem is approved for use in medicine as listed by the Ayurvedic Pharmacopoeia of India.[5] This is due to higher alkaloid content in the stems than in the leaves. It is a traditional belief that *Guduchi satva* obtained from the *Guduchi* plant growing on *neem* tree *(Azadirachta indica)* is more bitter and more efficacious and is said to incorporate the medicinal values of *neem*.[38]

## EFFECTS ON STRESS, LEARNING AND MEMORY

*T. cordifolia* is known as a *medhya rasayana* (learning and memory enhancer) in Ayurveda. It is also described to be useful for treatment of *bhrama* (Vertigo) in various Ayurvedic texts. Significant response has been found in children with moderate degree of behavior disorders and mental deficit, along with improvement in IQ levels.[34]

The root of *T. cordifolia* is known to be used traditionally for its anti-stress activity.[22] In a 21-day randomized, double-blind placebo-controlled study, the pure aqueous extract of the root was found to enhance verbal learning and logical memory.[39] *T. cordifolia* has also been shown to enhance cognition (learning and memory) in normal rats and reverse cyclosporine-induced memory deficit. Both the alcoholic and aqueous extracts of *T. cordifolia* produced a *decrease* in learning scores in Hebb William maze and retention memory, indicating enhancement of learning and memory. The histopathological examination of hippocampus in cyclosporine-treated rats showed neurodegenerative changes, which were protected by *T. cordifolia*.[40] Various extracts of the *T. cordifolia* exhibited comparable anti-stress activity in mice.[41,42]

## ANTI-INFLAMMATORY, ANTI-ARTHRITIC AND ANTI-OSTEOPOROTIC ACTIVITIES

*T. cordifolia* is mentioned to treat *vatarakta* (gouty arthritis) and *daha* (burning sensation) in various Ayurvedic texts [Table 2]. It is traditionally used in compound formulations for the treatment of rheumatoid arthritis.[43] The alcoholic extract of *T. cordifolia* has been found to exert anti-inflammatory actions in models of acute and subacute inflammation.[44] The water extract of the stem of *neem-giloe* [The *T. cordifolia* that grow on *Azadirachta indica (neem)*] significantly inhibited acute inflammatory response evoked by carrageenin in a dose of 50 mg/100 g given orally and intraperitoneally. A significant inhibition of primary and secondary phases of inflammation was observed in a model of adjuvant-induced arthritis. It also significantly inhibited antibody formation by typhoid "H" antigen. A mild analgesic effect of its own as well as potentiation of morphine analgesia has been reported.[45] In another study aqueous extract of *T. cordifolia* showed a significat antiinflammatory effect in the cotton pellet granuloma and formalin induced arthritis model, it's effect was comparable with indomethacin and its mode of action appeared to resemble that of nonsteroidal antiinflamatory ageant. The dried stem of *T. cordifolia* produced significant anti-inflammatory effect in both acute and subacute models of inflammation. *T. cordifolia* was found to be more effective than acetylsalicylic acid in acute inflammation, although in subacute inflammation, the drug was inferior to phenylbutazone.[46] The aqueous extract of stem was reported to exert a significant anti-inflammatory effect in both cotton pellet–induced granuloma (1, 250 and 500 mg/kg given orally) and formalin-induced arthritis (1 mg/kg given orally) rat models.[47,48]

## ANTI-ALLERGIC ACTIVITY

*T. cordifolia* is used for the treatment of *kasa* (cough) and *swasa* (asthma), which is described in various texts of Ayurveda [Table 2]. *T. cordifolia* is traditionally used for the treatment of asthma, and the juice is also employed for the treatment of chronic coughs.[49] In a clinical study, 100% relief was reported from sneezing in 83% of the patients on treatment with *T. cordifolia*,. Similary, there was relief from nasal discharge was reported in 69%; from nasal obstructions 61% and from nasal pruritus, in 71%. In placebo group, there was relief from sneezing only in 21% patients; from nasal discharge, in 16.2%; from nasal obstruction, in 17%; and from nasal pruritus, in 12%. Thus, *T. cordifolia* significantly decreased all symptoms of allergic rhinitis and was well tolerated.[50] The anti-allergic and bronchodilator properties of an aqueous extract of the stem evaluated on histamine-induced bronchospasm in guinea pigs, capillary permeability in mice and mast cell disruption in rats showed that it significantly decreased bronchospasm induced by 5% histamine aerosol, decreased capillary permeability and reduced the number of disrupted mast cells.[51,52]

## ANTIOXIDANT ACTIVITY

*T. cordifolia* is mentioned as *vishaghni, vishahara and tridoshashamaka* in various texts of Ayurveda [Table 2]. A significant increase in the concentration of thiobarbituric acid-reactive substances (TBARS) in brain, along with its decrease in heart, was observed in diabetic rats. *Tinospora cordifolia treatment* decreased the concentrations of glutathione reductase (GSH) and decreased activities of superoxide dismutase (SOD), catalase and glutathione peroxidase (GPx) in the tissues of diabetics rats. Alcoholic extract of the root of *T. cordifolia* (TCREt) administered at a dose of 100 mg/kg orally to diabetic rats for 6 weeks normalized the antioxidant status of heart and brain. The effect of *T. cordifolia* root extract was better than glibenclamide (600 µ/kg) although Insulin (6 units/kg) restored all the parameters to normal status.[53,54] *T. cordifolia* has also been reported to elevate GSH levels, expression of the gamma-glutamylcysteine ligase and Cu-Zn SOD genes. The herb also exhibited strong free radical-scavenging properties against reactive oxygen and nitrogen species as studied by electron paramagnetic resonance spectroscopy.[55] Aqueous extract of *T. cordifolia* inhibited Fenton (FeSO4) reaction and radiation - mediated 2-deoxyribose degradation in a dose-dependent fashion, with an IC50 value of 700 µ/mL for both Fenton and radiation-mediated 2-DR degradation. Similarly, it showed a moderate but dose-dependent inhibition of chemically generated superoxide anion at 500 µ/mL concentration and above, with an IC50 value of 2000 µ/mL.[56] In various studies, *T. cordifolia* was found effective in iron-mediated lipid damage and gamma-ray-induced protein damage,[57] amelioration of cyclophosphamide-induced toxicity,[58] alteration of lethal effects of gamma rays,[59] induction of enzymes of carcinogen/drug metabolism and inhibition of lipid peroxidation in mice,[60] free radical generation and lipid peroxidation during oxygen-glucose deprivation,[61] and nitric oxide scavenging effetcs.[62] The extract of *T. cordifolia* has demonstrated antioxidant action in the alloxan induced diabetes model as well.[63]

## ANTINEOPLASTIC AND RADIO-PROTECTIVE ACTIVITY

Intraperitoneal injection of the alcoholic extract of *T. cordifolia* has been shown to Dalton's lymphoma (DL) bearing mice e stimulated macrophage functions likephagocytosis, antigen-presenting ability and secretion of Interleukin-1 (IL-1), tumour necrosis factor (TNF) and Reference Nutrient Intake (RNI) as well as slowed tumor growth and increased lifespan of the tumor-bearing host.[64] *T. cordifolia* was has beem shown effective in several other tumour models including Ehrlich ascites carcinoma (EAC) in mice.[65] It induces proliferation and myeloid differentiation of bone marrow precursor cells in a tumor-bearing host,[66] activates tumor-associated macrophages-derived dendritic cells,[67] is effective against various cancers,[68] killing the cancer cells very effectively *in vitro*[69,70] inhibits skin carcinogenesis in mice,[71] and inhibits experimental metastasis.[72] *T. cordifolia* may offer an alternative treatment strategy for cancer in combination with gamma radiation.[73,74]

## ANTIPYRETIC AND ANTI-INFECTIVE ACTIVITY

Traditionally *T. cordifolia* is known for its *jwarahara* activity (antipyretic activity), as mentioned in Table 2. The water-soluble fraction of 95% ethanolic extract of *T. cordifolia* plant has shown significant antipyretic activity.[75] In another experimental study, antipyretic effects have been reported in the hexane- and chloroform-soluble portions of *T. cordifolia* stems.[76] Various studies show remarkable anti-infective and antipyretic properties of *T. cordifolia*.[77,78] Pre-treatment with *T. cordifolia* was shown to impart protection against mortality induced by intra-abdominal sepsis following coecal ligation in rats and significantly reduced mortality from induced by *E. coli*–induced peritonitis in mice.[79]

## HEPATO-PROTECTIVE ACTIVITY

Various Ayurvedic preparations of *T. corfifolia* are indicated in *pandu* (anemia) and *kamla* (jaundice). A clinical study has shown that *Guduchi* plays an important role in normalization of altered liver functions (ALT, AST).[80] The antihepatotoxic activity of *T. cordifolia* has been demonstrated in CCl_4_ induced liver damage, normallising liver function as assessed by morphological, biochemical (SGPT, SGOT, serum alkaline phosphatase, serum bilirubin) and functional (pentobarbitone sleep time) tests. *T. cordifolia* revealed hepatoprotective action in goats.[81] A significant increment in the functional capacities of rat peritoneal macrophages was observed following *T. cordifolia* treatment.[82] Addition of extract for the first 6 weeks to chloroquine showed regression of spleen by 37% to 50% after 6 weeks and 45% to 69% after 6 months from the start of treatment. Likewise, decrease in IgM and increase in Hb, as well as wellbeing (Karnofsky performance scale), were observed.[83] *T. cordifolia* prevents antitubercular drugs[84,85] and bile salts[86] induced hepatic damage, x and obstructive jaundice.[87] The extract has also exhibited *in vitro* inactivating property against hepatitis B and E surface antigens in 48 to 72 hours.[88]

## ANTI-HYPERGLYCEMIC ACTIVITY

*T. cordifolia* is widely used in Ayurveda for treating diabetes mellitus.[53,58,63] Various studies demonstrate amelioration of experimental diabetic neuropathy and gastropathy in rats,[89] reduction of blood sugar in alloxan-induced hyperglycemic rats and rabbits,[90] significant reduction in blood glucose and brain lipids,[91] increase in glucose tolerance in rodents,[92,93] increase in glucose metabolism,[93] inhibitory effect on adrenaline-induced hyperglycemia by pyrrolidine derivative,[94,95] and significant hypoglycemic effect in normal and alloxan diabetic rabbits[96] following administration of *T. cordifolia*.

## IMMUNOMODULATORY ACTIVITY

In Ayurveda *T. cordifolia* is believed to have rasayana (rejuvenating), *balya* (tonic), *vayah-sthapana* (anti-aging), *aayushyaprada* (increases the lifespan), *vrishya* (aphrodisiac) and *chakshusya* (useful in eye disorders) properties [Table 2]. The alcoholic and aqueous extracts of *T. cordifolia* are reported to have beneficial effects on the immune system[81,87] and have been tested successfully for their immunomodulatory activity.[97-103] The degradation of proteins due to photosensitization as assessed by Sodium dodecyl sulfate-polyacrylamide gel electrophoresis (SDS-PAGE) was effectively reduced by simultaneous treatment with G1-4A/PPI (partially purified immunomodulator) from *T. cordifolia* during photosensitization.[104] The novel (1,4)-alpha-D-glucan derived from the plant activates the immune system through the activation of macrophages *via* TLR6 signaling, NFkappaB translocation and cytokine production.[32,105] *Tinospora cordifolia* differentially regulate elevation of cytokines as evidenced by the increased production of antiangiogenic agents IL-2 and tissue inhibitor of metalloprotease-1 (TIMP-1) in the B16F10-injected, extract-treated animals. The observed antiangiogenic activity of the plant *T. cordifolia* is related to the regulation of the levels of cytokines and growth factors in the blood.[106] The aqueous extract of *T. cordifolia* was found to enhance phagocytosis *in vitro*. The aqueous and ethanolic extracts also induced an increase in antibody production *in vivo*.[107] *T. cordifolia* extract (TCE) treatment caused significant reduction in eosinophil count and improved hemoglobin in HIV patients. Sixty percent patients receiving TCE and 20% on placebo reported decrease in the incidence of various symptoms associated with disease.[108] Diabetic patients with foot ulcers on *T. cordifolia* as an adjuvant therapy showed significantly better final outcome with improvement in wound healing.[109] Administration of *T. cordifolia* (200 mg/kg body weight) 1 hour before irradiation showed recovery of spleen weight from 49% of control in irradiated group to 93%; apoptosis, from 19% to 2.8%; DNA fragmentation, from 43% to 20.4%; macrophage adherence, from 75% of control to 120%; and macrophage spread size, from 8 µ to 15µ. It also stimulated proliferation in splenocytes in a dose-dependent manner. Administration of *Tinospora cordifolia* (*Tc*) before irradiation also increased levels of IL-1beta and GM-CSF from 56 pg/mL and 53 pg/mL in irradiated group to 59 pg/mL and 63 pg/mL, respectively. Similarly, radiation-induced decrease of antioxidant potential of plasma [32 Fe(2+) equiv.] as compared to control [132 Fe(2+) equiv.] was countered by administration of *Tc* before irradiation [74.2 Fe(2+) equiv.]. RTc treatment thus suggesting its radio-protective mechanism.[110] Ten days of treatment with *T. cordifolia* (100 mg/kg/d) induced a significant (*P* < 0.01) increase in the number of (Colony Forming Units of ranulocyte-macrophage series (CFU-GM; 255 ± 49.32 *vs*. 38.51 ± 9.98). This suggests that activation of macrophages by *T. cordifolia* leads to increase in GM-CSF, which leads to leukocytosis and improved neutrophil function.[111]

## DIURETIC ACTIVITY

*T. cordifolia* has been described as useful in *mutrakriccha* (urinary trouble) separately and in the form of various formulations in Ayurveda, as mentioned in Table 2. In a scientific study on rats and human volunteers, *T. cordifolia* was found to have diuretic effects.[112] It was also found effective in modulation of morphology and some gluconeogenic enzymes activity in diabetic rat kidney.[113]

## CARDIOPROTECTIVE ACTIVITY

*Bhavprahash Nighantu* and *Shaligram Nighantu* describe *T. cordifolia* to have *hrudya* (cardioprotective) properties and is useful in *hridroga* (cardiac disorders) (Table 2). A dose-dependent reduction in infarct size and in serum and heart lipid peroxide levels were observed with prior treatment with *T. cordifolia* in ischemia-reperfusion–induced myocardial infarction in rats.[114] The stem extract has been normalize alterations in the lipid metabolism caused by diabetes mellitus in streptozotocin-induced diabetic rats indiectly benefiting the heart.[115] Administration of the extract of *T. cordifolia* roots (2.5 and 5.0 g/kg body weight) for 6 weeks resulted in a significant reduction in serum and tissue cholesterol, phospholipids and free fatty acids in alloxan diabetic rats.[116]

## ANTI-LEPROTIC ACTIVITY

*T. cordifolia* is used for its *kushtahara* (anti-leprotic) properties, along with wide use in *kandu* and *visarpa* (types of skin disorders) and has been shown to exert anti-leprotic activity in a combination formulation.[117]

## GASTROINTESTINAL AND ANTI-ULCER ACTIVITY

Ayurvedic properties of *T. cordifolia* include *sangrahi, arshahara, aruchinashaka, dipana, agnidipana, chardihara, trishnahara, trishnanashaka and hikkahara*. Treatment with a formulation containing *T. cordifolia* has been shown to reduce ulcer index total acidity, with an increase in the pH of gastric fluid in pylorus-ligated rats and in the ethanol-induced gastric mucosal injury in rats.[118]

## ANTIFERTILITY ACTIVITY

Oral administration of 70% methanolic extract of *T. cordifolia* stem to male rats at a dose level of 100 mg/d for 60 days did not cause body weight loss but decreased the weight of testes, epididymis, seminal vesicle and ventral prostate in a significant manner.[119]

## OSTEOPROTECTIVE ACTIVITY

Rats treated with *T. cordifolia* (10 mg/kg body weight) showed an osteoprotective effect, as the bone loss in tibiae was slower than that in controls. Serum osteocalcin and cross-laps levels were significantly reduced. This study demonstrates that extract of *T. cordifolia* has the potential for being used as antiosteoporotic agent.[120]

## SAFETY PHARMACOLOGY

It is a common misconception that Ayurvedic medicines are always safe. In fact, they also pose serious health risks either in the form of adverse reactions or in the form of drug interactions. In a clinical study, *T. cordifolia* has been shown to be at a dose of 500 mg/d for a period of 21 days in healthy individuals.[80] It has also been shown not to exert any remarkable adverse effects on the cardiovascular system,[121,122] renal system,[112,122] central nervous system[39-40,45,121,123] and gastrointestinal system.[49,101,124]

## CONCLUSION

The pharmacological actions attributed to *Tinospora cordifolia* in Ayurvedic texts have been validated by a remarkable body of modern evidence suggesting that this drug has immense potential in modern pharmacotheraoeutics.
